# Occupational Therapy and Public Safety Personnel: Return to Work Practices and Experiences

**DOI:** 10.1177/00084174231222075

**Published:** 2023-12-25

**Authors:** Megan Edgelow, Ana Petrovic, Clare Gaherty, Agnieszka Fecica

**Keywords:** Occupational health, Psychological trauma, First responders, PTSD, Premiers répondants, santé occupationnelle, traumatisme psychologique, TSPT

## Abstract

**Background.** Public safety personnel (PSP) are frequently exposed to psychological trauma through their work. Evidence shows that worker's compensation claims for work-related psychological injuries are on the rise for PSP. Occupational therapists increasingly provide return to work (RTW) services for this population. **Purpose.** To explore the therapeutic practices and personal experiences of occupational therapists working with PSP who have work-related psychological injuries. **Method.** This mixed methods descriptive study included a chart review of available occupational therapy client records from 2016 to 2020 for PSP with work-related psychological injuries from two Ontario companies. Additionally, a web-based self-report survey for Ontario occupational therapists providing RTW services to this same population was available from November 1, 2021 to June 1, 2022. **Findings.** The chart review included 31 client records and the online survey was completed by 49 Ontario occupational therapists. Therapists commonly provided services in clients’ homes, workplaces, and communities, and focused on functional activities. The evidence base drawn on by therapists was not always occupation-based. Barriers to RTW included challenges with interprofessional collaboration, stigma, and the COVID-19 pandemic. **Implications.** Occupational therapists are commonly working with PSP with work-related psychological injuries and have the opportunity to contribute to the evidence base for occupational approaches to RTW.

## Résumé

**Description.** Le personnel de sécurité publique (PSP) est fréquemment exposé à des traumatismes psychologiques en contexte de travail. Il a été démontré que les demandes d’indemnisation pour des lésions psychologiques liées au travail étaient en augmentation pour cette population. Les ergothérapeutes fournissent de plus en plus de services de retour au travail au PSP. **But.** Explorer les pratiques thérapeutiques et les expériences personnelles des ergothérapeutes travaillant avec des membres du PSP souffrant de lésions psychologiques liées au travail. **Méthodologie.** Cette étude descriptive à méthodes mixtes comprend un examen des dossiers de PSP ayant reçu des services d’ergothérapie pour des lésions psychologiques liées au travail entre 2016 et 2020 dans deux entreprises de l’Ontario offrant des services de réadaptation. En outre, une enquête d’auto-évaluation en ligne auprès des ergothérapeutes de l’Ontario fournissant des services de retour au travail à cette même population a été effectuée du 1^er^ novembre 2021 au 1^er^ juin 2022. **Résultats.** L’examen des dossiers a porté sur 31 dossiers de clients, et 49 ergothérapeutes de l’Ontario ont répondu à l’enquête en ligne. Les ergothérapeutes fournissaient généralement des services à domicile, sur le lieu de travail et dans la communauté, et se concentraient sur les activités fonctionnelles. La base de données probantes sur laquelle s’appuyaient les ergothérapeutes n’était pas toujours fondée sur l’occupation. Les obstacles au retour en emploi comprenaient les difficultés liées à la collaboration interprofessionnelle, la stigmatisation et la pandémie de COVID-19. **Conséquences.** Les ergothérapeutes travaillent souvent avec des membres du PSP souffrant de lésions psychologiques liées au travail et ont la possibilité de contribuer à la base de données probantes sur les approches occupationnelles du retour au travail.

## Introduction

“Public Safety Personnel” (PSP), which includes first responders, refers to professionals such as border services officers, communicators and dispatchers, correctional workers, firefighters, paramedics, police officers, search and rescue personnel, and others who work to ensure the safety and security of the Canadian public (Canadian Institute for Public Safety Research and Treatment, 2022). Due to the nature of their work, PSP are frequently exposed to traumatic events, potentially increasing their risk of developing mental health disorders, including posttraumatic stress disorder (PTSD), generalized anxiety disorder, and depression (Carleton et al., 2019). The symptoms of these conditions may include sleep disturbances, mood fluctuations, and cognitive disruptions, which can have a profound impact on an individual's quality of life and may necessitate taking time off work (Torchalla et al., 2019).

In Ontario, psychologically injured PSP can access care via the Workplace Safety and Insurance Board of Ontario (WSIB), which provides health care coverage, loss of earnings benefits, and return to work (RTW) support services. These claims can be costly at the individual, organizational, and systemic level. The direct annual costs of psychological distress in Ontario were estimated to be $441 million in 2013 (Chiu et al., 2017) while productivity losses alone were estimated to cost Canada $20.7 billion annually (Wilson et al., 2016). With an estimated “69,000 police personnel; 110,000 firefighters; 30,000 paramedics; 17,000 correctional services personnel; over 7,000 border services personnel; and 18,000 volunteer search and rescue personnel” at risk of psychological injuries in Canada, these costs can be substantial (Public Safety Canada, 2022).

In an effort to address this pressing issue, the Ontario government passed legislation in 2016 that presumes that PTSD in PSP is work-related, increasing access to mental health support for PSP. Since the introduction of this legislation, WSIB mental stress injury claims covering lost work time for this population increased from 0.3% in 2002 to 3% in 2020 (WSIB, 2020). Between January 1, 2016 and April 30, 2020, WSIB approved 5691 claims made by all PSP career categories under the presumptive PTSD legislation (Conference Board of Canada, 2021). A recent study of WSIB claims made between 2017 and 2021 revealed that the number of claims in Ontario has grown, with significantly more claims made in 2021 (n  =  1420) compared to 2017 (n  =  1050) (Edgelow et al., 2023a). This study also revealed that the majority of approved claims were made by police (33.5%), paramedics (28.4%), and correctional workers (21.6%). The remaining claims were made by firefighters (9.4%) and communicators (7.1%) (Edgelow et al., 2023a).

To date, little is known about the experiences of PSP who have made this type of WSIB claim. A recent survey-based study of 145 Ontario PSP who had an approved WSIB mental stress injury claim explored this experience with a focus on the RTW process (Edgelow et al., 2023b). The study revealed that the most frequently accessed healthcare providers were psychologists (61%), occupational therapists (60%), and general practitioners (44%). The study found that PSP rated their experience of WSIB and employer support as poor on their first RTW attempt. Respondents identified the cultural competence of healthcare providers in understanding their work culture and work demands as very important. However, this study did not document the details of the treatment these PSP received during the course of occupational therapy.

We do know from existing research that occupational therapists working with psychologically injured PSP may use a variety of approaches including psychoeducation, re-engagement in meaningful activities, a focus on routine and daily structure, teaching coping skills, and symptom management strategies (Torchalla et al., 2019); however, the extent to which these practices are used to provide RTW services to injured PSP is not yet known. Furthermore, we know that occupational therapists can work effectively in partnership with psychologists for RTW by extending the therapeutic approaches directly into the workplace, but little is known about the details of these collaborations (Edgelow et al., 2019, 2020; Gross et al., 2021). Despite the recent publication of the Occupational Therapy Trauma Intervention Framework, a readiness-based clinical reasoning framework developed by occupational therapists for occupational therapists working with clients who have experienced trauma (Edgelow et al., 2020), the degree to which the elements of this practice framework are used in practice is currently unknown.

Occupational therapists are frequently engaged by the WSIB to provide RTW services for PSP with work-related psychological injuries and the literature describing the practices and experiences of occupational therapists working with this population is limited. Thus, this study's main purpose was to explore the therapeutic practices and experiences of occupational therapists working with PSP clients who have made a WSIB claim due to a work-related psychological injury with a specific focus on RTW services.

## Method

### Study Design

A sequential exploratory mixed methods design (Creswell & Creswell, 2018) was used to document the practices and experiences of occupational therapists providing RTW services to psychologically injured PSP with two phases of data collection. The first phase of data collection involved the extraction of clinical data from clients’ charts which documented RTW services provided to psychologically injured PSP by occupational therapists employed by two Ontario-based rehabilitation companies. The second phase of data collection involved the distribution of an online survey to occupational therapists who provide RTW services to psychologically injured PSP in Ontario. The study received ethical clearance from the Queen's University Research Ethics Board (REH-781-20).

#### Phase 1: chart review

The goal of the chart review was to document the therapeutic practices of occupational therapists providing care to psychologically injured PSP in Ontario.

*Recruitment*: Convenience sampling was used for the chart review; program managers from two large Ontario-based rehabilitation companies known to provide RTW services to PSP were contacted by the lead researcher to understand the availability of data and amenability to participating in research. Following approval from the leadership of the companies, confidentiality agreements were signed, and data access was provided to the research team.

*Data collection and instrumentation*: The two companies provided the research team with remote access to their client charts which documented RTW services for psychologically injured PSP that had occurred between 2016 and 2020. Only client charts where the client (a) was a PSP and (b) received WSIB-funded RTW services for a work-related psychological injury were considered eligible for the chart review.

Eight key pieces of information were extracted from each eligible chart and captured in an Excel spreadsheet (Microsoft Corporation, Seattle, WA, 1985–2023) including the client's age, occupation, location, referral source, contextual information related to RTW processes, occupational therapy assessment and treatment practices, and length of time receiving RTW services.

*Data analysis*: Frequency counts and descriptive analyses were used for quantitative data, and framework analysis was used to analyze the narrative contents of clients’ charts. As a flexible applied qualitative research method, framework analysis provides a structured approach to real-life investigations that consists of sifting, charting, and sorting information, allowing for a blended inductive and deductive approach (Goldsmith, 2021; Ritchie & Spencer, 2002; Ward et al., 2013). The analysis was guided by Ritchie and Spencer's (2002) five key stages: familiarizing, identifying a thematic framework, indexing, charting, mapping, and interpreting. We used MAXQDA (VERBI Software Consult Sozialforschung GmbH, 1989–2023), a qualitative data analysis software program, to facilitate analysis within the team.

During familiarizing, clients’ charts were reviewed by three members of the research team. After becoming familiar with the contents of the charts, the Occupational Therapy Trauma Intervention Framework (Edgelow & Cramm, 2020) was identified as a useful base to build a thematic framework in order to interpret the data collected in the study. The Occupational Therapy Trauma Intervention Framework is an evidence- and theory-informed clinical reasoning framework that was developed in response to the absence of a guiding practice framework for occupational therapists working with clients who have experienced work-related psychological trauma. The framework was designed to support therapists’ clinical reasoning and match a client's level of readiness to engage with a spectrum of evidence-based OT approaches (Edgelow & Cramm, 2020). The framework incorporates enablement skills, assessment, treatment, and general approaches used by occupational therapists, derived from trauma research and occupational therapy theory. The Occupational Therapy Trauma Intervention Framework provides a foundation for the development of evidence-informed occupational therapy practice with clients who have experienced work-related psychological trauma (Edgelow & Cramm, 2020).

The Occupational Therapy Trauma Intervention Framework's intervention domains related to therapeutic approaches, assessment tools, and treatment methods (Edgelow & Cramm, 2020) formed the basis of the thematic framework. The research team then proceeded with indexing, charting, mapping, and interpreting the data. During the indexing phase, commonalities and consistencies within the data were noted. In charting, sub-categories of the thematic framework were created to capture the most consistent and common issues noted within the main categories. Finally, mapping and interpreting were informed by the content generated in the indexing and charting phase, as well as by existing literature.

#### Phase 2: survey

While the chart review explored the content of therapeutic approaches used by occupational therapists providing RTW services to PSP with work-related psychological injuries, it offered limited insight into occupational therapists’ experiences with this population, and only captured the practices of therapists working for two Ontario companies. Thus, the purpose of the survey was twofold. The first goal was to build on the findings of the chart review by further exploring the contents of the therapeutic approaches used by occupational therapists providing RTW services to psychologically injured PSP, capturing a broader group of therapists. The second goal of the survey was to explore occupational therapists’ personal experiences of providing these services.

*Recruitment*: The web-based self-report survey was distributed in English to occupational therapists in Ontario, Canada and was hosted on the Qualtrics platform (Qualtrics, Provo, UT, 2005–2023). Convenience sampling was used to recruit survey participants via professional associations (e.g., COTO and OSOT) and through the research teams’ social media accounts (e.g., Twitter and Facebook). In addition, anonymous links to the survey were emailed to employers and sole practice occupational therapists identified as WSIB service providers throughout Ontario.

*Data collection and instrumentation*: Any occupational therapist who provided RTW services to PSP with a work-related psychological injury in Ontario was eligible to participate in the study. Survey responses were collected from November 1, 2021, through June 1, 2022.

The survey instrument consisted of 43 closed- and open-ended items organized into 6 sections: demographics, work history and experience, occupational therapy professional development, occupational therapy practice competencies, access to occupational therapy services, and interprofessional collaboration. To maximize the survey's usability, questions related to assessment, treatment, and barriers and facilitators to RTW included pre-populated lists along with the option to add “other” items or comments. Open-ended items were also included in the survey to allow participants to provide more specific information on their practice experiences. The questionnaire was piloted with several occupational therapists who were not survey participants before it was made available to participants.

*Data analysis*: Frequency counts and descriptive analyses were used for quantitative data, and framework analysis was used to analyze the open-text participant responses. As in the chart review phase, Ritchie and Spencer's (2002) five key stages were used to guide analysis. During familiarizing, the survey responses were reviewed by three members of the research team. After becoming familiar with the contents of the surveys, the Occupational Therapy Trauma Intervention Framework (Edgelow & Cramm, 2020) formed the basis of the thematic framework for analysis of the survey data. The framework's intervention domains related to therapeutic approaches, assessment tools, and treatment methods were again used to inform the thematic framework that supported indexing, charting, mapping, and interpreting the data (Ritchie & Spencer, 2002).

## Findings

### Phase 1: Chart Review

*Participant characteristics***:** Of the 57 charts identified for review, 31 met inclusion criteria and represented clinical records from 2016 to 2020. At 58% of the total sample, paramedics comprised the biggest group of PSPs treated, followed by police officers (16%), communication officers (16%), and firefighters (10%). Most of the PSP treated were male (68%). Clients ranged in age from 23 to 63 years with most making a WSIB mental stress injury claim in their 40s (34%) or 50s (37%). The mean age of WSIB claimants was 46.6 years of age. See Table 1 for summary.

**Table 1:** Demographic Characteristics (Chart Review)

	Frequency (n = 31)	
Gender		
	Woman	32% (10)
	Man	68% (21)
Career		
	Communication officers	16% (5)
	Firefighters	10% (3)
	Paramedics	58% (18)
	Police officers	16% (5)
Age (in years)		
	Mean	46.61
	Range	23–63
	Standard deviation	9.40

*Occupational therapy practices***:** The most frequently used assessments focused on measuring symptom changes, posttraumatic growth, and resilience (e.g., Hospital Anxiety and Depression Scale; Posttraumatic Growth Inventory; PTSD Checklist). The chart review revealed that occupational therapists rely predominately on psychological, occupational, and coping skills approaches when providing RTW services to psychologically injured PSP. The most frequently used treatment methods included coping skills development, cognitive behavioural therapy (CBT), education, and exposure therapy. Many occupational therapists collaborated with several different healthcare professionals, with a third of the charts referencing some form of interprofessional collaboration during treatment.

*Barriers and facilitators***:** Analysis of clients’ charts revealed that family support was viewed by occupational therapists as an important RTW facilitator. Within the clinical chart data, the COVID-19 pandemic was frequently perceived as a barrier. Some occupational therapists noted that COVID-19 restrictions interfered with progress toward reintegration into more populated and varied locations. As a result, RTW could not be recommended until clients had an opportunity to interact in more diverse settings where their emotional state could be evaluated. In the clinical data, it was noted that some clients struggled with commitment and engagement in occupational therapy, including completing homework and demonstrating progress toward occupational therapy goals. Overall, most comments in the clinical data were respectful and person-centred in nature, however, there were some instances where the documentation was less recovery focused.

### Phase 2: Survey

*Participant characteristics***:** A total of 49 Ontario-based occupational therapists providing RTW services to PSP with a work-related psychological injury completed the survey. The majority (85%) of respondents identified as women and 58% fell between 25 and 34 years of age. The majority (80%) of respondents worked as an occupational therapist for fewer than 14 years with most (82%) providing occupational therapy services to PSP for fewer than 5 years. Respondents practiced in numerous settings including clinics (14%), via telehealth (23%), in the community (22%), in clients’ homes (20%), and in the workplace (20%). See Table 2 for summary.

**Table 2:** Demographic Characteristics (Survey Respondents)

	Frequency (n = 49)	
Gender		
	Woman	84% (41)
	Man	12% (6)
	Genderfluid	2% (1)
	Not reported	2% (1)
Age		
	20–24	2% (1)
	25–29	29% (14)
	30–34	29% (14)
	35–39	16% (8)
	40–44	12% (6)
	45–49	6% (3)
	50–54	4% (2)
	55–59	2% (1)
Race		
	White	84% (41)
	Asian	10% (5)
	Other	4% (2)
	Latin(a/o/x)	2% (1)
OT work experience (in years)		
	0–2	18% (9)
	3–4	20% (10)
	5–9	22% (11)
	10–14	20% (10)
	15–19	12% (6)
	20–24	0% (0)
	25–29	2% (1)
	30+	4% (2)
OT experience with PSP (in years)		
	0–2	51% (25)
	3–4	31% (15)
	5–9	10% (5)
	10–14	4% (2)
	15–19	2% (1)
	20–24	2% (1)
Practice setting (respondents could select more than one setting)		
	Clinic	14% (24)
	Telehealth/virtual	23% (40)
	In-home services	20% (34)
	Client's workplace	20% (34)
	Community	22% (39)
	In-patient program	2% (3)

*Occupational therapy practices***:** Like the chart review, the survey data revealed that the most frequently used assessments focused on measuring symptom changes, posttraumatic growth, and resilience (e.g., Hospital Anxiety and Depression Scale; Posttraumatic Growth Inventory; PTSD Checklist). A notable difference was observed between our two data sources, with survey respondents reporting occupation-based assessments such as the Canadian Occupational Performance Measure (COPM), time use diaries, and job demands analysis more frequently than the clinical records reviewed (Table 3).

**Table 3:** Occupational Therapy Assessment Approaches

Assessment	Frequency of use by OTs	
	Survey (n = 49)	Clinical Records (n = 31)
*Symptom-based assessments*		
Beck anxiety inventory (BAI)	38.78%	12.5%
Hospital Anxiety and Depression Scale (HADS)	42.86%	59.38%
Patient Health Questionnaire (PHQ-9)	48.98%	18.75%
PTSD Checklist (PCL-5/PCL-C)	77.55%	75.00%
*Functional/occupation-based assessments*		
Canadian Occupational Performance Measure (COPM)	77.55%	18.75%
Goal Attainment Scale (GAS)	24.49%	—
Job demands analysis	36.73%	—
Time use diary	28.57%	—
WHO Disability Assessment Schedule (WHODAS 2.0)	14.29%	3.22%
*Other assessments*		
Connor–Davidson Resilience Scale 25 (CD-Risc-25)	40.82%	40.63%
Posttraumatic Growth Inventory (PTG-I)	51.02%	56.25%
Readiness Ruler (RR)	28.57%	40.63%

The survey revealed that occupational therapists use a variety of therapeutic approaches when providing services to psychologically injured PSP including exposure therapy, behavioural activation, and forms of cognitive therapies. For instance, a survey respondent who primarily treats police officers, paramedics, and correctional officers relies on “behavioural activation and exposure [as] the main interventions” and “work[s] with clients in their client homes, communities (e.g., grocery stores), and workplaces.” Others noted that “the reason for referral is usually behaviour activation and exposure, however, they are always open to other treatment goals after I assess. Clients usually also require more attention to basic self-care, and coping skills development before they can increase their activities in the community or exposure therapy.” Respondents expressed tensions with respect to their autonomy in setting clients’ therapy goals: “there is internal pressure from our managers to always list exposure therapy and RTW as goals, even if I don’t think it's appropriate for where the client is at currently.”

Reiterating the findings of chart review, the survey revealed that occupational therapists rely predominately on psychological, occupational, and coping skills approaches when providing RTW services to psychologically injured PSP. The most frequently used treatment methods included coping skills development, cognitive behavioural therapy (CBT), education, and exposure therapy. However, in the area of productivity, survey respondents indicated that they used graded work exposure, cognitive work hardening, and connection to community resources. In contrast, the clinical data revealed a deeper focus on RTW planning and behavioural activation (Table 4).

**Table 4:** Occupational Therapy Treatment Approaches

Treatment type	Frequency of use	
	Survey (n = 49)	Clinical Records (n = 31)
*Psychological treatments*		
Acceptance and Commitment Therapy	12.24%	6.45%
Behavioural Activation	20.41%	28.13%
Cognitive Behavioural Therapy and techniques	81.63%	40.63%
Exposure therapy	85.71%	65.63%
*Occupational treatments*		
Cognitive work hardening	46.94%	—
Connection to community resources	59.18%	—
Education (Psychoeducation, education on SMART goals, etc.)	87.76%	71.88%
Graded work exposure	73.46%	—
Return to work planning	2.04%	28.13%
*Coping-focused treatments*		
Meditation	77.55%	3.13%
Relaxation	85.71%	53.13%
Self-regulation strategies	57.17%	31.25%

The survey data highlighted the unique contributions that occupational therapists make to the treatment of PSP with work-related psychological injuries. One such contribution involves the flexible nature of occupational therapy services with one respondent noting that: “OT's services are far more functional in nature; this is largely due to the service delivery model (i.e., OTs can attend locations throughout the community, employer, etc. to service client needs).” Another respondent shared: “we have the benefit of supporting the client from this lens as well as being in-vivo in the appropriate environments/contexts such as the workplace.” An additional respondent stated that “we provide direct, real-time support in the community assisting them to return to activities they need to do like going grocery shopping and to the pharmacy, leisure activities they want to do because it brings them joy, and activities they feel expected to do like work on household management or return to work.”

Another unique contribution made by occupational therapists included a focus on improving a clients’ functional abilities. Self-regulation training and activity-based interventions such as in vivo exposure therapy and facilitating community participation were used to increase clients’ overall participation and engagement in activities, often in community settings. The survey data echoed the findings of the chart review with many occupational therapists identifying activity-based interventions, working with clients in the community, and accompanying clients for in-vivo exposures as their unique contribution to RTW practices for PSP.

While the chart review data shed minimal light on the frequency of OTs’ interprofessional collaboration with other professionals, the survey data revealed more information. Respondents collaborated with psychologists (73.47%), WSIB case managers (65.31%), employers (44.90%), and psychiatrists (30.61%); however, many also reported frustrations with respect to these collaborations including communication difficulties between collaborators and WSIB case managers. In general, respondents expressed a desire for more interprofessional collaboration and reciprocated communication with professionals (particularly psychologists; Table 5).

**Table 5:** Interprofessional Collaborators (Survey Findings)

Professional	Frequency (n = 49)
Employer	44.90%
General practitioner (Physician)	22.45%
Nurse practitioner	8.16%
Other	12.24%
Physiotherapist	18.37%
Psychiatrist	30.61%
Psychologist	73.47%
Social worker	12.24%
WSIB case manager	65.31%

*Barriers and facilitators***:** The survey data revealed that clients’ social supports, employers, and healthcare professionals can all act as facilitators to RTW. Survey data showed that good communication with other healthcare professionals, positive collaboration with WSIB, and building strong rapport with clients by using humor were all important facilitators to occupational therapists’ work (Table 6). One respondent noted that WSIB RTW specialists “act as a liaison between the client and clinicians and employer and having that mediation is hugely helpful. They also sometimes know what to ask for specifically in a workplace to make accommodations feasible.”

**Table 6:** Barriers and Facilitators to RTW (Survey Findings)

Factor	Frequency (n = 49)		
	Facilitator (%)	Barrier (%)	Both (%)
Availability of service	8.16	6.12	18.37
Communication among members involved in the individual's care	12.24	8.16	28.57
Confidentiality	14.29	2.04	8.16
Elimination of stigma	16.33	6.12	6.12
Environment/setting of treatment	22.45	6.12	18.37
Experience with other healthcare professionals	12.24	0	34.69
Expertise of healthcare professionals	14.29	0	24.49
Expertise of WSIB case manager	14.29	8.16	22.45
Family responsibilities	0	8.16	14.29
General knowledge about services	14.29	30.61	8.16
Other	0	2.04	0
Policies	0	8.16	6.12
Socioeconomic status	0	12.24	12.24
Stigma	0	36.73	2.04
Support from leadership/employer	12.24	16.33	18.37
Support from peers/family members	18.37	2.04	22.45
System navigation	4. 08	30.61	8.16
Timing/scheduling	6.12	16.33	8.16
Transportation	2.04	4.08	14.29
Treatment outcomes/results	18.37	2.04	8.16

Respondents reported WSIB system navigation difficulty, stigma, lack of support from employers, communication difficulties, and the COVID-19 pandemic as barriers to RTW. Communication difficulties with other healthcare professionals and WSIB were perceived as especially challenging with one occupational therapist noting “How can we plan cohesive treatment and work on an effective recovery and RTW when all parties are not communicating? It's very challenging. We are all siloed and cannot communicate openly, regularly and freely.” Lack of perceived support from employers also impacted communication: “Clients are scared to have us talk to employers, they are suspicious of their employers’ intentions and fear reprisal when they RTW.” Others noted that they felt that WSIB did not fully understand the purpose of occupational therapy in the RTW process: “WSIB case managers often don’t know all that we can do and will refer people who are not engaged with any meaningful life roles for exposure therapy and RTW.” Finally, the costs associated with professional development and lack of evidence-based literature specific to occupational therapy interventions were also identified as barriers and areas of improvement required to provide better services to clients (Table 6).

*Experiences of occupational therapists:* Survey responses shed light on the experiences of occupational therapists providing RTW services to PSP with psychological injuries, including the importance of building rapport with clients, the challenging nature of the work and its impact on therapists. Respondents perceived dark humour and the use of clients’ language as critical tools to build rapport and connect with PSP during sessions: “Definitely humour and using the client's language, letting them feel comfortable swearing and with darker humour is necessary honestly.” An appreciation of work culture was also deemed vital: “I think the key in rapport building is demonstrating a knowledge of the culture; it's great if you know the language/terms/acronyms they use and how they use dark humour to cope.”

Some respondents reported finding the work of providing RTW services to PSP with psychological injuries emotionally taxing with the work impacting a therapist's own mental health and potentially leading to burnout. One respondent noted the “gradual/cumulative negative emotion dealing with the weight of stories impacting my own emotional state and behavioural reaction.” However, many respondents felt that the work, although difficult, can also been very rewarding.

## Discussion

Motivated by a growing number of WSIB mental stress injury claims made by PSP who often turn to occupational therapists for support, and a lack of existing research documenting occupational therapy with this population, the aim of the current study was to document the practices and experiences of occupational therapists providing RTW services to psychologically injured PSP in Ontario.

### The Focus of Occupational Therapy

Assessments used by occupational therapists ideally reflect the focus of the profession and maintain a person-centered, evidence-based, and occupation-focused approach to guide the treatment approach (Brown et al., 2019; Egan & Restall, 2022). This study found that occupational therapists providing RTW services for PSP with work-related psychological injuries used both symptom-based and occupational assessment approaches. Although a wide variety of symptom and occupation-focused assessments were used, a discrepancy in the use of occupation-focused assessments between survey data and clinical data was observed. Survey respondents reported using more occupation-focused assessments than were captured in the clinical data. Given that occupational therapy is rooted in improving occupational performance and increasing participation, this finding is particularly interesting (Egan & Restall, 2022). The primary focus of occupational therapy services in this study was occupation-focused, being that most clients were there to RTW. However, the clinical data revealed a stronger focus on assessing symptoms, which may not accurately represent a client's functional abilities. These findings echo those of a recent scoping review, which observed the use of occupation-focused assessments in the treatment of PTSD in a mere 17 out of 50 occupational therapy sources (Edgelow et al., 2019). The root cause of this finding is not fully understood and requires further research.

Given the central focus of the profession, we expected to find that occupational therapists primarily addressed their client's occupational performance and participation rather than impairments and symptoms (Beauchesne & Jacques, 2015; Edgelow et al., 2019). Specifically, we anticipated finding evidence of the use of empirically supported occupational therapy practices such as psychoeducation, occupational engagement, supported employment, time-use interventions, and skill development training (Kirsh et al., 2019). Other common intervention approaches for trauma and stressor-related disorders include trauma-informed care, therapeutic writing/creative media, mindfulness, and developing coping strategies (Brown et al., 2019). Unexpected differences were observed between the survey data and clinical records with survey respondents reporting psychological, occupation-based, and coping skills approaches evenly, while the practices documented in the clinical data focused primarily on psychological and coping skill treatments. In addition, clinical records also revealed that occupational therapists tended to perform exposure therapy and CBT by incorporating components of community integration or engagement/participation in psychotherapeutic approaches, allowing them to modify the treatment in real-time and support a client in overcoming barriers specific to their natural context (AOTA, 2020). Limited evidence, however, supports occupational therapists’ use of psychotherapy approaches with this population (Kirsh et al., 2019). Additionally, reviews of psychotherapeutic approaches for PTSD have revealed high dropout rates and a lack of measurement of functional outcomes, meaning that the evidence base for psychotherapy with PSP is not as strong as some may assume (Najavits, 2015; Sciarrino et al., 2020; Steenkamp et al., 2015). In contrast, previous studies have demonstrated that work-focused therapies have the highest efficacy for RTW in clients with common mental disorders, and that PSP clients value the practical and applied approach that occupational therapists can offer them in their RTW journeys (Edgelow et al., 2023b).

These findings raise questions about the part that occupational therapists play in the RTW process of psychologically injured PSP and whether the profession is used to its full potential. The chart review revealed a strong tendency for occupational therapists to use psychological treatment terminology over occupational terminology. The concern regarding the prevalent use of this terminology in occupational therapy documentation is that doing so may lead to confusion and a misunderstanding of occupational therapists’ RTW practices. Additionally, opportunities to advance the science of occupational therapy are missed when approaches and terminology from outside the profession are dominantly used. The reason for the prevalence of this terminology in the clinical data is not known but may be related to institutional factors such as company policies and procedures which impact treatment choices, as well as the terminology used in the WSIB referral process and by other healthcare professionals. On a few occasions, the clinical data explored in this study documented referrals for “exposure therapy.” Referrals for a specific psychotherapy intervention highlight that funding sources can limit services and entrenched practices reduce the autonomy of individual practitioners to formulate interventions based on their clinical judgment. The prevalence of biomedical and psychological models in Western medicine challenges occupational therapists in practicing a more holistic, relationship-centred, and occupation-focused model as healthcare systems focus on treating symptoms and pathologies rather than improving occupational performance and participation (Egan & Restall, 2022; Gentry et al., 2018). Occupational therapists have the opportunity to reframe psychological terminology from referral sources related to cognitive and exposure therapy to include language in their clinical documentation that is more relevant to occupational therapy, including terms like participation, occupational engagement, and community and work reintegration.

Since its inception, occupational therapy's distinct value in supporting mental health has been its use of activity and meaningful occupation as a therapeutic medium (AOTA, 2016; Brown et al., 2019; Freeman et al., 2022). This vital contribution is also highlighted in The Canadian Model of Occupational Participation (CanMOP) which explicitly states that occupation is the profession's primary concern (Egan & Restall, 2022). Using a functional lens separates occupational therapists from other healthcare professions who tend to use a biomedical or psychological model of treatment and focus more on the treatment of symptoms rather than occupational performance and participation (Egan & Restall, 2022; Freeman et al., 2022).

In this study, the Occupational Therapy Trauma Intervention Framework (Edgelow & Cramm, 2020) was used as a guiding theoretical framework. While elements of the framework were evident in the assessment and treatment practices captured in this study, its intervention domains should be more fully used by occupational therapists to ensure an occupation-centred approach (Edgelow & Cramm, 2020). Occupational therapists’ use of an occupational lens provides a unique focus that complements the work of other healthcare professionals and promotes client success in RTW. While many other healthcare professionals provide services exclusively in controlled clinical settings, occupational therapists work in environments such as the client's home, place of work, or community. Providing clinical services in natural and realistic settings allows the therapist to observe nuances elicited by the environment and to tailor interventions more effectively to a client's needs (Brown et al., 2019). The current study revealed that occupational therapists working with psychologically injured PSP frequently provided community-based services to their clients, with a recent study confirming this as an advantage of occupational therapy for PSP (Edgelow et al., 2023b).

### Barriers and Facilitators to RTW

A frequently referenced barrier to RTW in our study was stigma (self-stigma, stigma from employers and coworkers, and stigma from healthcare professionals). While the stigma surrounding mental illness in the general public is well documented, PSP can experience a unique type of stigma. PSP report experiencing more systemic stigma around mental illness rather than stigma about having the mental illness itself (Ricciardelli et al., 2020). Systemic stigma includes placing the workload of an employee seeking healthcare services on the remaining workers, thereby increasing their workload and potentially leading to coworker resentment (Ricciardelli et al., 2020). Additionally, while the general public experiences the potential for loss of status as a result of mental health stigma, PSP experience this as a particular concern given their occupation and workplace culture (Crowe et al., 2015).

Interprofessional collaboration is known to positively impact client health outcomes (Seaton et al., 2021); however, although empirical evidence supports the use of interprofessional collaboration, facilitating and executing effective interprofessional collaboration can often be challenging. Commonly documented barriers include perceiving other professions as rivals, not wanting to interact with professions who are considered of lower status, boundary disputes regarding overlapping roles, and the use of different theoretical models of practice (Green & Johnson 2015). Previous work has documented occupational therapists’ susceptibility to tensions with healthcare professionals who prefer biomedical or psychological approaches in traditionally biomedical settings (Egan & Restall, 2022). Our findings reiterate these tensions, with respondents reporting disruptions to collaboration due to hierarchical dynamics and delayed or absent communication.

This study highlights the faciliatory effects of several key factors in RTW including support from clients’ family and friends, employers, and healthcare professionals. Understanding the client's perspective on what constitutes a facilitator is critical and may impact the success of an intervention. For clients who receive occupational therapy services after a long leave due to mental health concerns, successful RTW can involve increased self-understanding (values, identity, and resources), positive encounters (feeling heard and taken seriously by healthcare professionals), and support from the surroundings (i.e., peers/significant others, employers, and the social welfare system) (Haugli et al., 2011). Mutual trust, a person-centred approach, contact and communication, and supportive social relationships are also facilitators to RTW (White et al., 2019). To direct future care most effectively, further research is required to understand the facilitators of occupational therapy RTW services for PSP.

### Considerations When Working With PSP

Providing RTW services to psychologically injured PSP can be a challenging experience for many therapists, requiring unique approaches. Several respondents reported relying on the use of dark humor and their client's language as important techniques to build rapport with clients. A recent scoping review also found that humor can facilitate rapport and ultimately lead to more successful therapeutic outcomes (Kfrerer et al., 2022). PSP have identified a strong desire to work with healthcare professionals who understand the nature of their work, their workplace culture, and the barriers they experience in RTW (Edgelow et al., 2023b). Occupational therapists can look to resources like the Canadian Institute for Public Safety Research and Treatment and PSP advocacy organizations to better understand the realities of PSP work and ensure their own cultural competency with PSPs.

Many respondents spoke of the psychologically taxing nature of providing services to PSP, citing burnout and other mental health impacts. This finding aligns with previous research documenting increased rates of burnout among occupational therapists working in mental health settings (Scanlan & Hazelton, 2019). In contrast, some survey participants also spoke of the rewarding nature of this work. Associating meaning with this type of challenging work may be a vital asset in promoting an occupational therapist's own mental health (Scanlan & Hazelton, 2019).

This study highlights the importance of occupational therapy's core person-centred focus. While PSP are referred to occupational therapy services for RTW, often, other meaningful areas of their lives have also been impacted. Occupational therapists collaborate with clients to align their goals with the goals of other interested parties by analyzing activities and identifying common skills needed to engage in work and self-identified meaningful activities successfully. By working to create meaningful occupation-based goals, clients can feel a sense of control and motivation to participate in treatment (Kessler et al., 2019).

### Study Limitations

This study made use of existing clinical records and novel survey data and was thus not able to report directly on client experiences or outcomes of WSIB claims. The research team was limited to the files provided by the two participating rehabilitation companies. PSP client files were searched manually as neither company had a specific mechanism for identifying them; as such, not all relevant PSP files may have been found. It is possible that study data is not representative of all WSIB RTW claims in Ontario for psychologically injured PSP, and that more recent clinical data may reveal different practices.

The survey was promoted by two occupational therapy associations, as well as several employers, and advertised through social media platforms. Since there is no publicly available list of occupational therapists in Ontario who work with the study population, the research team could not directly advertise to this group or calculate a response rate to the survey. Inevitably, some perspectives were not captured. Most respondents had practiced for fewer than 14 years total and fewer than five years with PSP; it is possible that a novice perspective was over-represented in the survey data.

The COVID-19 pandemic and associated activity restrictions began in March 2020. Clinical data included the year 2020, and the survey took place between the fall of 2021 and spring of 2022; it is possible that a study conducted after COVID-19 restrictions were lifted would produce different results.

### Implications for Practice and Future Research

The results of this study reveal that many occupational therapists incorporate psychotherapeutic approaches originating outside occupational therapy when working with PSP with work-related psychological injuries. Although psychotherapy modalities are frequently touted as best practices, most studies of their efficacy focus on symptoms as a source of outcome measurement, and their evidence often stops short of functional measures (Ennis et al., 2021; Jericho et al., 2022). Further, research related to common mental disorders like anxiety and depression shows that symptom reduction alone is often not adequate to return clients to work, requiring functional approaches to bridge the gap to the workplace (Axen et al., 2020; Edgelow et al., 2023b; Torchalla & Strehlau, 2018). Additionally, a recent study of Ontario PSP with work-related psychological injuries reported valuing occupational therapy's practical and applied approach to RTW and their access to community environments linked to workplaces (Edgelow et al., 2023b). Together, this evidence serves as encouragement for occupational therapists to maintain the profession's core focus on occupation.

Survey respondents expressed a desire for more occupation-based professional development opportunities and more published research surrounding occupational therapy-specific RTW interventions to facilitate evidence-based practice. In response, occupational therapy professional associations should seek out occupation-focused professional development opportunities for their members and ensure that available research funding focuses on occupation-based research. Occupational therapists must advocate for and embody their profession's core values of person-centredness and occupation-focused practice, particularly in healthcare settings that emphasize biomedical and psychological models of practice. Occupational therapy education programs must ensure that occupation-focused practice is at the core of their curricula, and accreditation bodies can play a role in supporting this focus. Although research on occupation-based mental health interventions exists (e.g., Kirsh et al., 2019) and the Occupational Therapy Trauma Intervention Framework (Edgelow & Cramm, 2020) provides a framework for trauma-focused occupational therapy practice, there is a need for further research exploring the use of occupation-focused interventions and their effectiveness with this population.

## Conclusion

Occupational therapists increasingly provide RTW services to PSPs with work-related psychological injuries. This study explored the practices and experiences of these occupational therapists. The results of our study revealed opportunities for occupational therapists to use occupational approaches to both assessment and treatment and to document and study these approaches. Barriers to RTW included challenges with interprofessional collaboration as well as stigma in PSP workplaces. Survey respondents expressed a desire for more published research surrounding occupational therapy-specific RTW interventions to facilitate evidence-based practice. Future studies should explore the use of occupation-focused interventions, their outcomes, and effectiveness for PSPs with work-related psychological injuries.

## Key Messages

- Occupational therapy assessments and treatments for PSP with work-related psychological injuries should ensure a focus on clients’ occupational performance and participation.
- Occupational therapists can bring a unique occupation-focused and community-based approach that may be especially useful to this population in their RTW journeys.
- Further research needs to be conducted exploring effective occupational therapy interventions for PSP returning to work after psychological injuries.
